# Extraocular features of Leber hereditary optic neuropathy: A scoping review

**DOI:** 10.14440/jbm.2024.0113

**Published:** 2025-05-14

**Authors:** Layla Ali, Iyawnna Hazzard, Niloufar S. Tehrani, Ubaid Ansari, Adam Ali, Preyasi Kumar, Nadia Ali, Gurkiranjeet Gakhal, Forshing Lui

**Affiliations:** 1 Office of Research, College of Medicine, California Northstate University, Elk Grove, California 95757, United States of America; 2 Department of Translational Science, Rush University Medical Center, Chicago, Illinois 60612, United States of America

**Keywords:** Genetics, Leber hereditary optic neuropathy, Systemic involvement, Treatment

## Abstract

**Background:**

Leber hereditary optic neuropathy (LHON) is a rare inherited mitochondrial disease that leads to mitochondrial dysfunction, resulting in optic nerve damage and vision loss. Systemic involvement has been reported in several LHON cases, referred to as LHON+ disorders. However, the causes and presentations of such conditions have been poorly studied. It is suggested that 90% of mitochondrial dysfunction is caused by one of three primary point mutations in mitochondrial DNA that affect respiratory complex I (referred to as mtDNA LHON), with unresolved cases of LHON being caused by other variants, known as autosomal recessive LHON. The cardiac, musculoskeletal, neurological, and auditory systems are commonly affected in LHON. For example, hypertrophic cardiomyopathy and sudden cardiac death have been linked to specific mutations. Neurological effects – such as dystonia, epilepsy, polyneuropathy, and ataxia – as well as hearing loss, have also been observed in patients with specific mitochondrial mutations. These findings highlight the need for a more comprehensive evaluation beyond standard ophthalmic assessments. LHON is typically diagnosed based on a combination of ophthalmic imaging, patient age and gender, clinical course (bilateral, rapidly progressive, and sequential visual loss), family history, maternal inheritance, and fundus appearance. However, the advent of genetic testing has significantly expanded the recognized phenotype. In terms of treatment, idebenone is the only FDA-approved therapy for LHON; however, intravitreal gene therapy yields promising improvement, especially for the most common m.11778G>A mutation, which accounts for 70% of causative mutations. At present, these therapies are confined to ocular treatment.

**Objective:**

This review highlights the importance of recognizing systemic manifestations of LHON, which are frequently overlooked in clinical practice.

**Conclusion:**

Early detection of these systemic manifestations, especially in cardiac and neurological systems, could help with prompt intervention and improve patient outcomes. Further research into gene therapy and mitochondrial replacement techniques holds promising potential for developing more effective treatment strategies.

## 1. Introduction

Leber hereditary optic neuropathy (LHON) represents a genetically inherited disease that generally afflicts young adult males and results in severe visual impairment.^1^ It is the most common inherited mitochondrial disorder.^1^ Most LHON cases are caused by mutations in mitochondrial DNA (mtDNA) and follow a maternal (mitochondrial) inheritance pattern.^1^ This is because male mtDNA, localized in the tail of sperms, does not contribute to the fetal mtDNA during fertilization. The majority of mitochondrial proteins and enzymes are encoded in the nucleus and are therefore, transmitted in an autosomal recessive (AR) manner.^2^,^3^ In contrast, the mitochondrial genome only contains 37 genes and encodes a minority of the mitochondrial proteins relative to the nucleus.^2^ Despite this, mitochondrial inheritance is 10 times more common than AR inheritance for variants that cause LHON.^3^

The three most common mtDNA variants associated with the LHON phenotype are m.11778G>A (*MT-ND4*), m.14484T>C (*MT-ND6*), and m.3460G>A (*MT-ND1*).^4^,^5^ On the other hand, other variants, such as AR LHON (also referred to as arLHON), have been reported as the cause of unresolved LHON cases, including *NDUFS2, MCAT*, and *NDUFA12*.^6^ A notable arLHON mutation leading to nuclear dysfunction of the DNAJC30 protein – a chaperone protein involved in the repair and maintenance of mitochondrial complex I – has been identified in more than 20% of LHON patients.^7^ Sex-related phenotypic differences in LHON expression demonstrated that males exhibit a higher percentage of the phenotype compared to females.^7^ Incomplete penetrance of the LHON allele may be attributed to the ratio of mutant-to-wild type mitochondria, a concept that can be explained by heteroplasmy – which refers to the coexistence of two or more genetic subpopulations of mitochondria within the same organism. In cases involving m.1178G>A, individuals with a mutational load higher than 60% are at a higher risk of developing optic neuropathy.^8^

Determining whether the causative mutation is due to arLHON or mtLHON in a clinical setting is relatively difficult due to their similar symptom profiles. In the pre-symptomatic phase, the eye typically appears normal in both appearance and function. However, with retinal examination, clinicians often observe a triad of circumpapillary telangiectatic microangiopathy, vessel tortuosity of the central retinal vessels without leakage on fluorescein angiography, and the hallmark subacute phase swelling of the retinal nerve fiber layer (RNFL). This is followed by the atrophic chronic phase with thinning of the RNFL due to retinal ganglion cell (RGC) and axonal degeneration. During this period, LHON patients generally experience bilateral sequential centrocecal scotoma over the course of a few months, with a minority recovering some vision more than 1 year after the onset of vision loss. This vision loss usually occurs in the third or fourth decade of life, though it rarely takes place after this age.

A reduction in ATP production by mitochondria, caused by dysfunction of complex I, has been suggested as the pathophysiological mechanism underlying vision loss. This leads to the discontinuation of RGC energy production and a shift to anaerobic metabolism. In addition, an increase in the level of reactive oxygen species results in DNA and cellular damage, leading to RGC apoptosis.^9^ Mitochondria play a crucial role in oxidative phosphorylation within the electron transport chain, generating ATP necessary for cellular energy production. Therefore, any mitochondrial cytopathy impairs energy production in cells and tissues. In LHON, increased oxidative stress in RGC leads to RGC dysfunction, with ascending optic neuropathy and atrophy,^1^ leading to the classic LHON phenotype of vision loss.

However, due to abnormal cellular energy production, other tissues with the highest oxidative metabolic demands – such as the brain, heart, skeletal muscles, and the cochlea of the inner ear – are also preferentially affected.^5^

Different mitochondrial disorders exhibit varying involvement patterns across various tissues and organs. However, all of these disorders have the potential to affect multiple organs, leading to systemic diseases. LHON predominantly affects the visual pathways, involving RGC and causing damage to the optic nerve, following chronic, end-stage damage, optic atrophy results. While other syndromic involvements are present, they are often under-recognized. This comprehensive scoping review aimed to investigate the multisystem involvement of LHON and provide insight into how the disease may manifest in other organs. Such understanding might assist with earlier detection of this condition and improve treatment efficacy.

## 2. Detection and diagnosis of LHON

Since LHON is a mitochondrial genetic disorder, patients with this disease can have various manifestations and clinical presentations. The hallmark feature of LHON is vision loss. Individuals with LHON usually display symptoms such as loss of central vision, blurred vision, and reduced color perception when they are young adults.

The presentation of vision loss in LHON typically begins between 15 and 35 years of age, involving painless yet severe asynchronous bilateral loss of central vision within weeks or months.^10^ Typically, the unaffected eye is impacted 3 – 6 months after the onset of the disease. In 25% of cases, the disease begins bilaterally.^10^ Vision loss can be classified into three phases: The pre-symptomatic, acute, and chronic phases.^6^ The manifestation of vision loss can differ based on the causative mutation. In addition, the last phase may involve either a late spontaneous improvement of vision or progression into a chronic phase. Spontaneous improvement of vision occurs when the edema in the peripapillary RNFL recedes. However, progression into the chronic phase is marked by optic atrophy and lack of visual recovery.^12^

LHON exhibits incomplete penetrance, though various factors can influence the likelihood of vision loss, such as a close family history or exposure to cigarette smoke and excessive alcohol consumption.^11^ On the other hand, asymptomatic carriers can present with different types of dyschromatopsia, depending on the mutation. For instance, arLHON with the *MCAT* mutation presents with tritan color defects (blue–yellow), whereas arLHON with the *NDUFS2* mutation manifests as protan or deutan defects (red–green), and there is no specific dyschromatopsia associated with arLHON involving the *DNAJC30* mutation. However, mtLHON mutations can cause red–green color vision deficiency.^6^

Due to the rarity of LHON, many doctors may instead suspect and begin to treat other causes of vision loss.Therefore, careful attention to the timeline of vision loss, along with proper genetic and visual testing, is essential for accurate diagnosis. Fast and accurate detection and diagnosis are crucial for genetic counseling and initiating personalized treatment.^9^

LHON is typically diagnosed using specialized ophthalmic imaging.^13^ This includes specialized ophthalmologic testing to examine the fundus and identify recognizable changes in the optic disc. In patients with LHON, visual field tests would typically show centrocecal scotomas.

Other diagnostic tools include optical coherence tomography, which is used to identify patterns of RNFL loss throughout the progression of LHON and to measure the ganglion cell layer thickness.^14^ Macular RGC loss is often found before the onset of clinical disease. Carriers of the mutation may be asymptomatic; their fundus examination results may be normal, or their optical coherence tomography angiography assessments may include abnormalities in the optic vasculature, such as telangiectatic vessels and microangiopathy, hyperemia of the optic disc, and increased thickness in the inferior and temporal quadrants of the RNFLs.^14^

Mitochondrial disorders may either occur due to *de novo* mutations or through maternal inheritance. Thus, a thorough family history could provide insight that may support the diagnosis. Ultimately, genetic testing confirming the causative mutation is essential for establishing the diagnosis of LHON. Over 90% of LHON mutations are caused by mtDNA point mutations at positions 11,778, 3,460, and 14,484.^1^

Sanger sequencing and next-generation sequencing are the common methods used for genetic testing. However, these methods require advanced instruments and skilled technicians, which may not be readily accessible in all settings and can take several weeks to produce results. This may delay treatment for individuals who need prompt attention for this disease.^16^

Recent advancements in genetic testing and molecular assays have been developed to facilitate more rapid diagnosis of the disease.^17^ These low-cost, rapid genetic tests are becoming more readily available and have been incorporated into public health systems, such as the United Kingdom National Health Service. For example, among patients with inherited ophthalmic diseases, 1 in 300 people in the United Kingdom carries a pathogenic mtDNA variant; however, not all carriers present with symptoms or clinical signs of LHON. Thus, testing must be interpreted within the clinical context.^17^

Approximately 70% of LHON cases are due to a substitution of G to A at position 11,778, which eliminates a SfaNI restriction enzyme recognition site and creates a MaeIII site. Assays have been developed for the presence of this mutation using these sites.^18^ Detection of this mutation in human mtDNA can be done using polymerase chain reaction amplification and agarose gel electrophoresis. These methods require a small amount of blood from the patient and are quick to perform, making them useful in diagnosing LHON.^19^

The CRISPR/Cas12a-based DNA detection method can also quickly, conveniently, and accurately detects and diagnoses the three LHON mitochondrial variants.^15^ This method requires only a single drop of blood, and the results can be determined within 30 min after sampling. This is particularly useful for identifying LHON patients with any of these three common variants; however, methods to analyze the entire mtDNA sequences would be required for the remaining 10% of LHON patients who do not have one of these variants.^18^

## 3. Multi-system involvement

In multiple case cohorts or case reports, various systemic manifestations have been reported, ranging from cardiac, neurological, endocrine, otologic, renal, and musculoskeletal symptoms.^20^ Overall, while mtLHON mutations may not have been sequenced in all patients, studies suggest a significantly greater involvement of mtDNA mutations compared to arLHON mutations.

### 3.1. Cardiac and skeletal muscle involvement

In recent years, various studies have analyzed data of patients to suggest various cardiac pathologies associated with all three major mitochondrial gene mutations in LHON. Hypertrophic cardiomyopathy, dilated cardiomyopathy, and Wolff–Parkinson–White syndrome are just a few of the conditions that have been reported.^20^,^22^ As a result, patients diagnosed with LHON due to visual defects are recommended to undergo cardiac evaluation using echocardiography (ECG).

Even when asymptomatic, a significant proportion of patients with mtDNA mutations m.11778G>A (*ND4*), m.3460G>A (*ND1*), and m.14484T>C (*ND6*) present with cardiac abnormalities. The Reference Center for Rare Diseases in Ophthalmology conducted a study on a cohort of 73 patients with LHON in Paris, France. A total of 17 patients exhibited abnormal echocardiograms (ECGs), including two patients presenting with Wolf–Parkinson–White syndrome, seven patients with short PR interval, one with large T-waves, and one with asymptomatic myocardial ischemia.^21^ All 17 of these patients had either m.11778G>A or m.3460G>A mutations and were asymptomatic. Another patient presented with cardiac complaints before ECG.^4^

Another study conducted in London, the United Kingdom, yielded similar findings in a cohort of 24 patients: 11 patients with the m.3460G>A (*ND1*) mutation, eight patients with m.11778G>A (*ND4*), and five patients with m.14484T>C (*ND*6). In this study, myocardial hypertrophy was only observed in patients with the m.3460G>A variant; however, cardiac symptoms were present across all three mutation types.^22^ In addition, cardiac abnormalities have also been identified in siblings of affected individuals who did not exhibit visual impairment.^23^ A Danish case report described a 53-year-old male patient with progressive blindness, hypertrophic cardiomyopathy, sinus rhythm, and short PQ interval, whereas his 54-year-old sister – without any visual symptoms – were presented with identical ECG findings as well as septal and apical hypertrophy, chronotropic incompetence, and sinus bradycardia.^23^ The patient’s children were obligate carriers of their mutual m.3460G>A (*ND1*) mutation, yet remained asymptomatic at ages 34 and 37.^23^

Other cardiac abnormalities associated with LHON include increased aortic stiffness and non-compaction. A study in 2008 involving 19 patients compared them with gender- and age-matched controls, revealing a significantly increased aortic stiffness index and diastolic aortic diameter in patients with any of the three LHON mutations (*ND1, ND4*, and *ND5*).^24^ However, stiffness is not a consistent finding, as one case report of a patient with the *ND4* variant described a patient with neurological and dilated cardiomyopathy symptoms instead.^25^ These variable phenotypes may suggest different etiologies for these cardiac symptoms. Nevertheless, hypertrophy or dilation has been observed in different patients with LHON.

Awareness of cardiac abnormalities associated with LHON-related mutations is particularly important as several case reports have been published regarding life-threatening cardiac conditions, including sudden cardiac death, as highlighted in the aforementioned London study.^22^ Additional case reports have described left ventricular hypertrabeculation in individuals associated with m.3460G>A (*ND1*) mutation, with or without Wolff–Parkinson–White syndrome.^26^,^27^ This phenotype is typically diagnosed using imaging modalities, such as transthoracic echocardiography, and is particularly concerning due to possible arrhythmias, thromboembolism, and heart failure.^28^ Excessive left ventricular hypertrabeculation, also referred to as non-compaction, may present in LHON as a lone symptom,^29^ or as part of a broader range of symptoms.^30^

Some studies also suggest that the cardiac manifestation in LHON may be due to the heteroplasmy rate of the abnormal mitochondria within the affected organ tissue. However, this hypothesis remains disputed.^20^,^31^

In addition to cardiac manifestations, several musculoskeletal associations have been reported in LHON, such as myoclonus^32^ and dystonia.^33^ Both conditions have been observed in case reports of LHON patients, although both myoclonus and dystonia are regarded as neurological dysfunctions as opposed to structural issues within the muscle fibers themselves. A microscopic study examining the extraocular muscle structure in LHON patients observed a significantly larger and more abundant number of mitochondria (mean diameter of 0.85 μm) compared to controls (mean diameter of 0.65 μm). These mitochondria were observed to fill the sarcoplasmic reticulum and occupy two-thirds of the cytoplasm in muscle fibers, even distorting the myofibrils.^34^ It has been suggested that the increased number of mitochondria, also known as mitochondrial biomass, could serve as a protective mechanism in response to decreased mitochondrial efficiency resulting from mutations in the mitochondrial respiratory chain complex I. Although the correlation between mitochondrial mass and clinical manifestation remains unexplored, this correlation should be further investigated for its predictive probability.^35^

### 3.2. Auditory, psychological, and neurological involvement

LHON, along with other mitochondrially inherited conditions such as maternally inherited diabetes and deafness, are commonly associated with hearing loss.^36^ Multiple studies have identified auditory and neurological deficits in patients with LHON; however, the results remain inconsistent. Hearing loss is not considered a common, frequent observation in patients with LHON. For instance, a study involving 10 patients with LHON reported no hearing impairment in any of the participants based on topographic neuro-auditory assessments of the middle and inner ear, suggesting that hearing loss is not a common symptom in LHON.^37^ However, other studies have identified an association between symptomatic LHON and hearing impairment.

In a prospective study evaluating auditory function in patients with LHON, absent auditory brain response, and decreased amplitude-modulation detection were observed, consistent with auditory neuropathy (AN).^38^ The decreased visual acuity – often leading to blindness or severe vision impairment in LHON – was correlated with the findings of auditory assessments of the patients. Patients with the poorest auditory outcomes also exhibited the worst visual acuity.^38^ In addition, a significant impairment in speech perception was observed in LHON patients compared to controls (*p*<0.01).^38^

In addition, several case studies have documented hearing impairment in LHON patients with specific mtDNA mutations. In a study conducted by Ceranic and Luxon,^39^ two patients with LHON exhibited asymmetric hearing loss due to AN.^39^ Both patients had a positive mt.11778G>A mutation with decreased or absence of the stapedial reflex, indicative of disruption of the auditory pathway and/or compromised conduction at the brainstem or cochlear nerve. Previous literature reviews identified the mt.11778G>A mutation in LHON to be associated with AN spectrum disorder.^40^ Other mtDNA mutations associated with auditory involvement include mt.14484T>C, mt.3460G>A, mt.3394T>C, and mt.4640C>A mutations,^38^ as highlighted in Table 1. These findings were suggested to result from neural abnormalities in the central auditory pathways.^16^ Due to these inconsistent findings, future investigative studies evaluating whether a specific genetic association exists between LHON and hearing impairment are warranted.

**Table 1:** Conditions of Leber hereditary optic neuropathy

System	Pathology	*MT-ND6*	*MT-ND4*	*MT-ND1*	*MT-ND2*	*ND6, ND4,* and *ND1*	*ND4* and *ND1*
Otologic	Auditory involvement^38^	+	+	+	+	-	-
Neurological	Migraine with or without aura^50^	+	-	-	-	-	-
	Temporal lobe epilepsy^43^	-	+	-	+	-	-
	Pharmacoresistant temporal lobe epilepsy^43^,^24^	-	+	-	+	+	-
	Psychomotor regression, refractory epilepsy, and progressive neurological abnormalities in the setting of normal muscular function^25^	-	+	-	-	-	-
	Posterior reversible encephalopathy syndrome^44^	-	+	-	-	-	-
	Demyelinating polyneuropathy^45^	-	+	-	-	-	-
	Cerebellar ataxia and peripheral neuropathy^25^,^29^	-	+	-	-	-	-
	Myoclonus^32^	-	+	+	-	-	-
	Widespread demyelinating lesions in the central nervous system^46^	-	-	-	-	-	-
	Drug-resistant epilepsy^41^	-	-	-	-	+	-
	Cerebellar ataxia^48^	-	-	-	-	-	+
Psychiatric	Bulimia nervosa, compulsions, psychogenic non-epileptic seizures^32^	-	+	-	-	-	-
Cardiac	Dilated cardiomyopathy^25^	-	+	-	-	-	-
	Aortic stiffness^24^	-	-	-	-	+	-
	Lone noncompaction^29^	-	-	-	-	+	-
	Myocardial hypertrophy^32^	-	-	-	-	-	+
	Left ventricular hypertrabeculation^20^	-	-	-	-	-	+
Musculoskeletal	Muscular cramps^41^	-	+	-	-	-	-
Endocrine	Hashimoto thyroiditis^43^	+	+	-	-	-	-
	Hyperthyroidism^53^	-	+	-	-	-	-
	Pituitary adenoma^49^	-	+	-	-	-	-
Renal	Chronic renal failure^56^	-	+	-	-	-	-

Notes: “+” indicates the presence of symptoms; “-” indicates the absence of symptoms.

In addition to the auditory involvement observed in LHON, several studies have reported associations between LHON and various neurological and psychological disorders, including myoclonus,^32^ epilepsy,^41^-^43^ encephalopathy,^44^ demyelinating polyneuropathy,^45^ widespread demyelinating lesions,^46^ cerebellar ataxia and peripheral neuropathy,^47^,^48^ as well as psychiatric disorders, including but not limited to bulimia nervosa, psychogenic non-epileptic seizures, and compulsive behaviors (Table 1).^32^ These disorders, similar to the auditory involvement in LHON, have also been associated with specific mtDNA mutations, particularly those affecting the mitochondrial nicotinamide adenine dinucleotide hydrogenase (*MT-ND*) enzymes.

Specifically, psychiatric disorders are associated with *MT-ND4* mutations,^32^ whereas neurological disorders bear an association with *MT-ND2*,^4^ *MT-ND6*,^46^,^50^ and *MT-ND4*^25^,^27^,^43^,^45^ mutations. Nevertheless, *MT-ND4* mutations appear to be the most common mtDNA mutation associated with neurological involvement in LHON. In particular, case reports have identified two LHON patients with pharmacoresistant temporal lobe epilepsy linked to underlying *MT-ND2* and *MT-ND4* mutations.^4^ *MT-ND4* mutations have also been observed in other epileptic disorders, including refractory epilepsy^42^ and drug-resistant epilepsy.^41^ Other neurological disorders observed in LHON patients with *MT-ND4* mutation included psychomotor regression,^42^ myoclonus,^42^ progressive neurological abnormalities,^42^ posterior reversible encephalopathy,^22^ cerebellar ataxia,^48^ and demyelinating polyneuropathy.^43^ In the case of cerebellar ataxia, the patient exhibited both the *MT-ND4* and mt.3394T>C mutations, suggesting that both genetic abnormalities may contribute to the development of an atypical LHON with neurological involvement.^48^ Finally, widespread demyelinating lesions in the central nervous system, outside the visual system, in combination with aggravating autoimmune pathologies, such as Hashimoto thyroiditis,^43^ migraines with or without aura, and transient neurological or visual disturbances,^50^ have been associated with *MT-ND6* mutations in LHON patients.

It is well-established that neurons, including RGCs and retinal nerve fibers, are cells with one of the highest metabolic rates, along with muscle cells,^51^ and they fundamentally rely on mitochondria to perform their functions.^52^ The brain, eyes, and auditory system contain an abundance of neurons, and thus, they are exclusively dependent on mitochondrial ATP generation. Therefore, disruption of mitochondrial metabolic activity could help explain the systemic pathogenesis of LHON. Future investigations should explore whether correcting specific mitochondrial mutations is directly associated with the extraocular manifestations of LHON and could potentially identify new treatment strategies using precision medicine.

### 3.3. Endocrine and renal involvement

While autoimmune conditions such as Hashimoto thyroiditis have been associated with the onset of LHON in the setting of widespread demyelinating lesions in the central nervous system, other endocrine pathologies have also been reported, including pituitary adenoma and hyperthyroidism.^20^

The presence of a pituitary adenoma in LHON was reported in a 24-year-old male with the m.11778G>A mutation.^49^ The patient initially experienced bilateral painless, subacute vision loss, which progressed to central scotoma and optic atrophy.^49^ Although this finding may be coincidental, it is suspected that compression of the pituitary adenoma on the optic chiasm could be a triggering factor in the manifestation of the LHON phenotype due to further damage of axoplasmic transport and increased stress on optic nerve pathways.^54^

Another endocrine manifestation associated with LHON is hyperthyroidism.^53^ A 39-year-old female patient was reported with bilateral vision loss and the m.11778G>A mutation.^53^ The patient also presented with abnormally elevated free thyroxine 4 levels, and her visual acuity improved following the normalization of these hormone levels. The exact pathophysiology remains unclear, though it is suspected that elevated circulating thyroid hormone levels are associated with increased metabolism, including feedback mechanisms and lipid metabolism.^54^ The mitochondria, therefore, play a crucial role in protecting the body from this hyperthyroid state by coordinating tissue oxidative stress responses and subsequent survival mechanisms such as apoptosis.^55^ Thus, while LHON may not directly cause hyperthyroidism, mitochondrial damage in LHON could be exacerbated by a hyperthyroid state, with possible improvement once the thyroid levels are normalized. However, given the singular nature of this resolution, we suspect that this case may represent an outlier in the typical clinical course of LHON.

Renal involvement is also a rare manifestation of LHON and is associated with the m.11778G>A mutation.^56^ In one case, a 27-year-old male patient presented with bilateral optic atrophy in addition to chronic renal failure.^56^ While the underlying mechanism remains unclear, it is possible that the high metabolic demands of the kidney contribute to mitochondrial dysfunction. This dysfunction plays a key role in disrupting cellular redox potential, leading to cell death and a progressive inflammatory response, thereby contributing to the progression of chronic kidney disease.^57^

## 4. Treatment strategies and areas for research

At present, LHON lacks a definitive cure; however, current treatment avenues, such as idebenone, have shown promising potential in improving visual outcomes in certain patients. Idebenone is a safe and well-tolerated drug approved by the European Medicines Agency in 2015.^58^ It is currently the only FDA-approved and clinically proven treatment for LHON. By bypassing the defective mitochondrial complex I, idebenone enhances the energy supply to RGC, thereby preventing further vision loss and promoting recovery.^58^ It is particularly effective when treatment starts soon after the onset of symptoms. A case series involving four LHON patients with the 11778G>A mutation in the *MT-ND4* gene reported outcomes following genetic testing and ophthalmologic examination.^43^ An 82-year-old male with a long history of vision loss was not considered for idebenone treatment. Two siblings, a 40-year-old male and a 31-year-old female, were treated with idebenone; however, only the woman showed positive outcomes, and both were lost to follow-up after a year.^59^ The fourth patient was a 46-year-old male diagnosed during the subacute stage. He initially deferred idebenone treatment, which allowed his visual field defects to progress. After eventually starting the medication, he demonstrated visual improvement after 17 months of treatment, which continued for 36 months with good results.^59^ This study highlighted the variability in response to idebenone treatment, with notable improvement in a female patient despite delayed diagnosis. Other studies have also demonstrated this variability, with limited data on its impact in treating the extraocular manifestations of this disease.^60^,^61^

Potential future areas for research include exploring gene therapy to correct mtDNA mutations and stem cell therapy to regenerate damaged optic nerve cells. One study assessed 16 LHON patients in preparation for a gene therapy clinical trial,^62^ evaluating visual function parameters over 12 months. Among the 32 eyes studied, 24 remained relatively stable in terms of best-corrected visual acuity (BCVA), whereas eight showed significant changes.^62^ Of these, five demonstrated improvements, whereas three demonstrated deteriorations. Changes in BCVA were significantly correlated with the visual field index, mean defect, and visual evoked potential. This study suggests that the effectiveness of LHON gene therapy should be primarily evaluated based on visual acuity, with additional considerations for visual field, RNFL thickness, and electrophysiology.^62^ The RESTORE study, a long-term follow-up of the RESCUE and REVERSE phase 3 trials, assessed the efficacy and safety of intravitreal lenadogene nolparvovec gene therapy for LHON in patients with the m.11778 G>A mutation.^63^ Among the 61 patients who were followed for up to 52 months after vision loss, there was a progressive and sustained improvement in BCVA. The final mean BCVA showed significant recovery. In addition, vision-related quality of life, measured using the VFQ-25 score, increased by seven points.^63^ This study concluded that the positive treatment effects observed 2 years after treatment in RESCUE and REVERSE trials were maintained at 3 years in the RESTORE trial, with a maximum follow-up of 52 months after patients began losing their vision.

The REFLECT phase 3 study evaluated the efficacy and safety of bilateral versus unilateral intravitreal injections of lenadogene nolparvovec gene therapy in LHON patients with the m.11778G>A mutation.^64^ The main goal of this study was to compare the improvement in visual acuity in the second-affected or not-yet-affected eyes treated with gene therapy versus placebo. Although the change in BCVA from baseline did not show a statistically significant difference at 1.5 years, significant improvements in BCVA were observed in eyes treated with intravitreal lenadogene nolparvovec in both bilateral and unilateral treatment groups.^64^ A higher proportion of patients in the bilateral treatment group achieved on-chart vision in one or both eyes at 1.5 years. The treatment was well-tolerated, with intraocular inflammation being the most common side effect, effectively managed with topical corticosteroids. Overall, the study supports a more favorable benefit-risk profile for bilateral injections compared to unilateral injections in improving BCVA in LHON patients.^64^

While this review offers valuable insight into the multisystem involvement of LHON, there remain several limitations, including the small sample size of several studies and variability in diagnostic criteria. Future research should aim to standardize diagnostic approaches and further explore the relationship between mitochondrial mutations and systemic manifestations.

## 5. Conclusion

LHON is a rare mitochondrial disease, and while optic neuropathy is a classic phenotype, systemic manifestations are poorly studied and may be more common than previously thought. Vision loss in LHON can be classified into pre-symptomatic, acute, and chronic phases, and the specific mutation responsible for a patient’s LHON can lead to varying presentations of this vision loss. Systemic involvement is more commonly associated with mtLHON compared to arLHON. Similar to vision loss, different causal mutations can lead to various systemic presentations. Although there is no cure for LHON, understanding the genetic causes and pathophysiology behind each mutation could be key in detecting and predicting the clinical course of the disease.

## Data Availability

Not applicable.
